# Hormonal Regulation of Response to Oxidative Stress in Insects—An Update

**DOI:** 10.3390/ijms161025788

**Published:** 2015-10-27

**Authors:** Dalibor Kodrík, Andrea Bednářová, Milada Zemanová, Natraj Krishnan

**Affiliations:** 1 Institute of Entomology, Biology Centre, Academy of Sciences, Branišovská 31, 370 05 České Budějovice, Czech Republic; E-Mails: bednarovaandrea@centrum.cz (A.B.); milada.zemanova@prf.jcu.cz (M.Z.); 2 Faculty of Science, University of South Bohemia, Branišovská 31, 370 05 České Budějovice, Czech Republic; 3 Department of Biochemistry, Molecular Biology, Entomology and Plant Pathology, Mississippi State University, Mississippi State, MS 39762, USA; E-Mail: nkrishnan@entomology.msstate.edu

**Keywords:** adipokinetic hormones (AKH), AKH gene, anti-oxidative mechanisms, insect endocrine system, FoxO, free radicals, insecticide, oxidative stress, signaling pathway

## Abstract

Insects, like other organisms, must deal with a wide variety of potentially challenging environmental factors during the course of their life. An important example of such a challenge is the phenomenon of oxidative stress. This review summarizes the current knowledge on the role of adipokinetic hormones (AKH) as principal stress responsive hormones in insects involved in activation of anti-oxidative stress response pathways. Emphasis is placed on an analysis of oxidative stress experimentally induced by various stressors and monitored by suitable biomarkers, and on detailed characterization of AKH’s role in the anti-stress reactions. These reactions are characterized by a significant increase of AKH levels in the insect body, and by effective reversal of the markers—disturbed by the stressors—after co-application of the stressor with AKH. A plausible mechanism of AKH action in the anti-oxidative stress response is discussed as well: this probably involves simultaneous employment of both protein kinase C and cyclic adenosine 3′,5′-monophosphate pathways in the presence of extra and intra-cellular Ca^2+^ stores, with the possible involvement of the FoxO transcription factors. The role of other insect hormones in the anti-oxidative defense reactions is also discussed.

## 1. Introduction

It is known that various chemical (pesticides, drugs, metals, smog, abnormal oxygen concentration, *etc.*), physical (radiation, temperature, noise, vibration, *etc.*), and physiological (diseases, injury, aging, inflammation, *etc.*) stressors can result in a stress situation that may upset functional homeostasis. One such situation is termed oxidative stress (OS), and is characterized by enhanced production of reactive oxygen species (ROS) with the simultaneous impairment of their scavenging systems. Increased concentrations of ROS result in oxidative damage to proteins, lipids, and nucleic acids, and thus functions of cells, organs, or the whole organism may be seriously disrupted, leading to death. To avoid or at least reduce this, organisms have developed effective defense systems controlled by nervous and endocrine centers. In mammals, the stress response pathways are controlled from the hypothalamic–pituitary brain center with the help of a suite of corresponding hormones. In insects, the anti-OS reactions seem to be regulated predominantly by adipokinetic hormones (AKHs) which are secreted from the corpora cardiaca—a small neuroendocrine gland connected with the brain. The present review summarizes the current knowledge on the role of AKH in activation of anti-OS reactions in insects published within the last decade.

## 2. Reactive Oxygen Species and the Phenomenon of Oxidative Stress

Oxygen is essential for aerobic life processes, yet, about 1%–3% or more of the oxygen utilized for respiratory purposes is converted to ROS such as O_2_^−^•, H_2_O_2_, and •OH by univalent reduction of oxygen [1]. Thus, ROS are generated by aerobic living organisms during the course of normal cellular metabolism. ROS, when generated at low to moderate concentrations, function in various physiological cellular processes, but at high concentrations can lead to adverse effects on cellular components such as modifications of lipids, proteins, and DNA [2]. The cytotoxic effects of ROS are in general neutralized by a complex array of enzymatic and non-enzymatic scavenging and detoxification mechanisms, which have evolved to combat the deleterious effects of ROS. As such, there is always a dynamic equilibrium between ROS production and its removal by powerful anti-oxidant systems. However, when the balance between ROS production and anti-oxidant defenses shifts in favor of oxidants then the phenomenon of “oxidative stress” is encountered, which contributes to many pathological conditions in the aerobic organisms. Hence, OS can be defined as a state in which oxidation exceeds the antioxidant systems in the organism, secondary to a loss of balance between them. As mentioned before, such deregulation of cellular homeostasis results in events such as lipid peroxidation, protein damage, oxidative damage to DNA, and also physiologic adaptation phenomena and deregulation of intracellular signal transduction.

### 2.1. Endogenous Sources of Reactive Oxygen Species (ROS)

ROS can be categorized into two groups—free radicals and non-radicals. Free radicals are generated when molecules end up having one or more unpaired electrons that make them reactive in an effort to attain stability. On the other hand, non-radical forms are generated when two free radicals share their unpaired electrons. In this review, we shall touch upon only three major ROS that are of physiological significance *viz*. superoxide anion radical (O_2_^−^•), hydroxyl radical (•OH), and hydrogen peroxide (H_2_O_2_). The major site for the production of the superoxide anion radical is the mitochondria. During aerobic respiration for generation of adenosine triphosphate (ATP), electrons are transferred through the mitochondrial electron transport chain for reduction of oxygen to water. However, during this process 1%–3% of all electrons leak from the system and produce superoxide anion radicals [1]. Superoxide anion radicals are also generated during phagocytic activity by leukocytes, monocytes, and macrophages that have microbicidal activity. Superoxide dismutases (SODs) convert the superoxide anion radical (O_2_^−^•) to hydrogen peroxide (H_2_O_2_). The hydrogen peroxide that is generated can easily diffuse across the plasma membrane. Hydrogen peroxide is also endogenously generated by xanthine oxidase, amino acid oxidase, and NAD(P)H oxidase [3,4] in peroxisomes. The Haber–Weiss (1) and Fenton reactions (2) also result in the breakdown of H_2_O_2_ to OH^−^ in the presence of Fe^2+^ or Cu^2+^ [5]:

Fe^3+^ + O_2_^−^ → Fe^2+^ + O_2_ (1)

Fe^2+^ + H_2_O_2_ → Fe^3+^ + OH^−^ + •OH (2)

Additionally, O_2_^−^• by itself can also react with H_2_O_2_ and generate OH^−^ [6,7].

### 2.2. Exogenous Sources of ROS

There are several exogenous oxidants that impact an organism and lead to the generation of ROS within their cells. A few pertinent sources of such oxidants have been elaborated below.

Ionizing radiation: In the presence of oxygen, ionizing radiation can convert hydroxyl radicals, superoxide anion radicals, and other organic radicals to hydrogen peroxide and organoperoxides. Reaction of these hydroperoxide species with redox active metal ions, such as Fe and Cu via Fenton reactions, leads to induction of OS [8,9].

Exposure to ozone: Exposure to ozone can lead to lipid peroxidation, since short-term exposure causes release of inflammatory mediators such as myeloperoxidase, eosinophil cationic proteins, and also lactate dehydrogenase and albumin [10].

Heavy metal ions from pollutants: Heavy metal ions such as iron, copper, cadmium, mercury, nickel, lead, and arsenic can induce the generation of ROS and lead to cellular damage. An important mechanism of metal-catalyzed free radical generation is via the Fenton-type reactions described above. Other than the Fenton and Haber–Weiss reactions, certain metal ions can interact directly with cellular molecules to generate free radicals, such as thiol radicals, or induce cell signaling pathways. These radicals may also react with other thiol molecules to generate O_2_^−^•. Further, O_2_^−^• is converted to H_2_O_2_, which causes additional oxygen radical generation.

Pesticides: Extensive literature suggests that ROS are continuously generated from exposure to environmental contaminants such as herbicides and insecticides [11,12,13].

## 3. Oxidative Stress and Antioxidant Responses in Insects

Insects, because of their ability to colonize various ecological niches, are exposed to multiple environmental stressors across a variety of habitats. Thus, insects, like other organisms, are subject to OS upon exposure to herbicides and insecticides [14] and also upon feeding (especially phytophagous and hematophagous insects) [15,16]. Also, low temperature thermal fluctuations and exposure to UV also promote ROS and lead to OS in insects [17,18]. To combat the effects of ROS, insects have evolved a suite of antioxidant defense mechanisms that involves both enzymatic and non-enzymatic components [19]. However, the regulatory mechanisms behind the triggering and sustained activity of the antioxidant systems are largely unknown. Insect neurohormones are one of the prime candidates that could play such regulatory roles [20,21]. The ensuing paragraphs deal with the current knowledge on such neurohormonal regulation of response to OS in insects.

## 4. Insect Hormones: A Brief Overview

The insect endocrine system represents the most sophisticated and well-developed humoral system controlling bodily functions among the invertebrates. Its main axis is created by two hormonal centers—(a) neurosecretory cells in the brain with the corpora allata and corpora cardiaca, and (b) prothoracic gland—accompanied by several groups of separate endocrine cells (in the neural ganglia, gut, gonads, and epidermis). This endocrine system synthesizes and secretes three major groups of insect hormones and biologically active factors [22]: ecdysteroids, juvenile hormones, and neurohormones.

(1) Ecdysteroids are produced mainly by prothoracic glands generally localized in the prothorax, and partially also by gonads, the epidermis, and several other tissues. Ecdysteroids are involved in the regulation of principal events in insect life such as development, molting, metamorphosis, and reproduction. In addition, they also participate in a number of diverse processes. As apparent from their names, ecdysteroids are steroid hormones.

(2) Juvenile hormones (JHs), are synthesized in a retrocerebral gland called the corpora cardiaca; they play a major role in the regulation of insect metamorphosis (by preventing its premature commencement), and in the control of reproduction by stimulation of gonadal development and vitellogenin synthesis (control of vitellogenin gene expression). Interestingly, JHs also regulate processes such as insect behavior, diapause, caste determination, and various polyphenisms [22,23]. JHs belong to a family of acyclic sesquiterpenoids.

(3) Neurohormones are produced by modified neurons specialized for secretory functions. These neurons are mostly localized in the brain, but also occur in other ganglia of the insect nervous system. Neurohormones are peptidergic compounds that regulate principal behavioral, physiological, and biochemical events in the insect body, including those described above for JHs and ecdysteroids [24]. The classification and nomenclature of neurohormones are not uniform, but according to their functions they are usually divided into metabolic, developmental, reproductive, myotropic, and chromatotropic neurohormones.

One of the best investigated groups of insect hormones is the so-called adipokinetic hormone/red pigment-concentrating hormone family (AKH/RPCH family). AKHs are metabolic neurohormones that trigger defense reactions and are responsible for maintaining homeostasis in the insect body by controlling anti-stress reactions, including those responsible in counteracting OS. Their characteristics are discussed in the following section.

## 5. Adipokinetic Hormones—Main Stress Hormones in Insects

Adipokinetic hormones (AKHs) are products of a neuroendocrine gland—the corpora cardiaca—connected with the brain. They are synthesized by specialized neurosecretory cells, and stored and released from the corpora cardiaca when necessary. AKHs comprise eight to 10 amino acids [24] and their sub-cellular signaling pathways are well documented in the fat body [25] (for details, see Section 5.3).

It is generally accepted that the main role of adipokinetic peptides is in the control of insect energy metabolism. Nevertheless, AKHs are pleiotropic in their functions, with a lot of activities in addition to their metabolic role. In general, AKHs act as typical anti-stress hormones: they mobilize lipid, carbohydrate, and amino acid energy stores by stimulating catabolic reactions to gain energy. Simultaneously, AKHs inhibit synthetic reactions; thus the mobilized energy is used to eliminate imminent stress situations and is not wasted on reactions that are not as important in such situations and could draw on energy if allowed to continue. These basic anti-stress reactions are followed by stimulation of physiological anti-stress response [26] that involves activation of heart beat [27] and general locomotion [28], enhancement of immune responses [29], regulation of starvation-induced foraging behavior of *Drosophila* [30], participation in the activation of the antioxidant mechanisms [21,31], and, as recently found, enhancement of food intake and digestive processes in insect gut [32,33,34].

### 5.1. Role of Adipokinetic Hormones (AKH) in the Insect Anti-Oxidative Stress Response

It is generally accepted nowadays that AKHs play an active role in the protection of insects against OS [21]. A number of papers published in the last decade (see below) documented the fact that oxidative stressors raise the level of AKHs in the insect body, and furthermore that application of external AKHs reduce OS markers experimentally increased by application of the stressors. This suggests the existence of a feedback regulation between the oxidative stressor and AKH activities, and, as such, these hormones are involved in the activation of insect antioxidant mechanisms.

Firstly, application of the oxidative stressors to the insect body increases the level of AKHs in the hemolymph. This effect was recorded for a redox cycling herbicide, 1,1′-dimethyl-4,4′-bipyridylium dichloride hydrate (paraquat) [31,35], which is commonly used to elicit OS conditions in the insect body, and also for a common OS metabolite hydrogen peroxide [36]. A similar effect was also recorded for the *Bacillus thuringiensis* toxin, *Galanthus nivalis* agglutinin [31], and several insecticides [37,38]; however, this topic is discussed in a separate section (see Section 5.2). The intensity of the level of AKH elevation in hemolymph varied slightly depending on the type of oxidative stressor or the extent of exposure to stressor, and often is species specific. For example, in the firebug *Pyrrhocoris apterus* application of paraquat increased the AKH titer in hemolymph about 5-fold 4 h after treatment [35], hydrogen peroxide in the same species increased the AKH level about 4-fold 24 h after treatment [36], and paraquat again in the Colorado potato beetle *Leptinotarsa decemlineata* increased the level about 2.7-fold (again 4 h after application) [31].

Similar variable results were also recorded when the AKH level was measured in the corpora cardiaca or in the whole CNS (central nervous system = brain with the corpora cardiaca attached). In the Colorado potato beetle, *L. decemlineata*, application of paraquat elevated the AKH level by 1.9 times [31]. On the other hand, treatment of *P. apterus* by paraquat elicited no effect on the AKH level in the CNS (both 4 h after the injections) [35], but application of hydrogen peroxide elicited a 2.8-fold increase [36]. Nevertheless, the differences between the AKH levels in the CNS and hemolymph evoked by stress are not so unexpected, because the total amount of AKH in those two body parts differs significantly: e.g., in *P. apterus* it was recorded that there is about 200 times the amount of AKH in CNS that there is in hemolymph [39], and additionally there is no positive correlation between them [39]. This result is in accordance with the assumption that the coupling between biosynthesis and release of the AKHs in synthetic cells of the corpora cardiaca is very weak or perhaps does not even exist [40].

An active role implicating the involvement of AKHs in the control of anti-oxidative defense mechanisms emanated from the effect of oxidative stressors on AKH level in insect CNS and hemolymph, which were documented by experiments demonstrating direct involvement of AKHs in the modulation of OS biomarker levels. Both enzymatic and non-enzymatic markers have been studied in relation to AKH activities so far. Among the enzymatic ones, a duo—catalase (CAT) and SOD—of free radical-scavenging enzymes standing as the first line of organismal defense against oxidative injury [41,42,43] were primarily investigated. It has been shown in *Spodoptera littoralis* larvae that expression of *CAT* and *SOD* genes was significantly elevated (more than doubled) 12 h after tannic acid feeding [44], which indicates involvement of transcriptional regulation of those enzymes during OS. The enhancement of expression of those genes is in agreement with results obtained from other organisms, where expression of *SOD* and *CAT* genes was enhanced during the OS elicited by different stressors [45,46]. Interestingly, the gene expressions were significantly reduced to the control level when the tannic acid feeding was followed by AKH treatment, which clearly indicates involvement of the AKH in the control mechanism. Surprisingly, activity of both SOD and CAT was not correspondingly stimulated by the tannic acid feeding; also, AKH alone showed no direct effect. These results indicate a complex regulation suggesting involvement of additional controlling factors that might cooperate with AKH action. On the other hand, one can speculate that the suppressive effect of exogenous AKH on *SOD* and *CAT* gene expression after tannic acid feeding might be the result of lowered OS induced by other AKH actions (decreasing protein carbonylation, increasing activity of other enzymes, *etc.*) rather than by a direct effect of AKH on the level of mRNA. Further, subsequent experiments, wherein brains (CNS) dissected from *P. apterus* adults were incubated *in vitro* in the presence of hydrogen peroxide or AKH or both [36], suggested a different mechanism of action for AKH: incubation with hydrogen peroxide significantly reduced CAT activity, whereas co-incubation with hydrogen peroxide and AKH together returned the activity level to the control.

The last enzyme studied was glutathione-*S*-transferase (GST), wherein it was observed that activity dropped significantly in the *S. littoralis* larvae fed on tannic acid but returned to the control level after co-treatment with AKH [44]. GSTs are a heterogeneous group of detoxifying enzymes with a broad range of substrate specificity including products of oxidative metabolism like quinones, carbonyls, and organic hydroperoxides [47,48]. It has been documented that GSTs are significantly inhibited by a number of inhibitors including those structurally similar to tannic acid (caffeic acid, gallic acid) [49]. Taken together, all these results indicate that AKH is somehow actively involved in the control of the SOD, CAT, and GST in insects; however, the mechanism remains unclear, being probably species specific and/or accompanied by other effects or factors.

It has been observed that AKHs can also affect non-enzymatic OS markers such as glutathione (GSH), protein carbonylation, lipid peroxidation, or total antioxidant capacity. One of the most important OS metabolites is GSH, a typical low molecular weight antioxidant. It is a thiol commonly occurring in the cytosol and other aqueous phases of diverse living organisms [50,51]. The GSH level in the hemolymph of experimental insects was significantly elevated (almost two-fold) after injection of AKH but was significantly reduced (by one-half or one-third) after paraquat application in both *P. apterus* [35] and *L. decemlineata* [31] hemolymph. Nevertheless, co-injection of AKH with paraquat resulted in the GSH level returning to the control. A slightly different effect was obtained in *S. littoralis*, where application of AKH elicited no significant effect in intact insects, and similarly, the same application elicited no relevant effect in GSH level significantly reduced by tannic acid feeding [44]. Thus, it seems evident that AKH plays a significant role in the release of reduced GSH into the insect hemolymph, but the effect seems to be species specific or varies according to the specific anti-oxidative response pathway activated by a corresponding stressor.

Another important biomarker of OS is protein carbonylation, whose level documents the amount of oxidative damage and hence reflects the anti-oxidative capacity of the organism. Carbonyls are formed from amino groups in the side chain of some amino acids that are exposed to ROS [52]. Protein carbonyls represent a sensitive and widely used OS marker: application of chemical stressors (paraquat, hydrogen peroxide, tannic acid) significantly elevated the protein carbonyl level in insect hemolymph, but their co-application with AKH reduced the carbonyl levels to those found in controls [31,35,36]. It should be mentioned that injection of AKH alone did not change the content of carbonyl in experimental insects. This suggests that a stressor action is probably necessary for AKH to exert its response by curbing formation of carbonyls. Indeed, numerous examples exist to show that the effect of AKH in the insect body is manifested only in the presence of a stressor: a positive correlation between the hyperlipaemic effect of AKH and its stimulation of locomotor activity was recorded in *P. apterus* only when AKH was applied via injection but not when AKH was applied topically. This implies that the stress of injection was necessary for the effects of AKH to be measurable. Further, injection of Locmi-AKH-I into *Locusta migratoria* significantly activated phenoloxidase activity only in the presence of laminarin (a glucose polysaccharide) or bacterial lipopolysaccharide, both considered stressors to the insect; when the agents were missing, no effect of AKH was observed [29]. Moreover, the effect of insecticides and AKHs on the intensity of insect metabolism monitored by carbon dioxide production [37,38,53] (for details see Section 5.2) also belong to this category. A mechanism explaining a role of these stressors in triggering the adipokinetic response is not yet known. However, it seems logical that at least in the case of injury, the nervous system might be involved [54]; nevertheless, in the absence of direct information, the possible involvement of other players (e.g., biogenic amines) in the cascade remains a speculation.

The listing of AKH actions in its anti-OS role also includes the control of lipid peroxidation in the cell membranes [36]. Incubation of *P. apterus* CNS with hydrogen peroxide *in vitro* revealed a similar data pattern to that mentioned above: a significantly higher level of lipid peroxidation in CNS in the presence of hydrogen peroxide alone, and a reduced level (comparable to controls) in the presence of hydrogen peroxide with AKH. Interestingly, application of AKH alone reduced the lipid peroxidation level to even below control values. This phenomenon begs a question: Does AKH prevent lipid peroxidation at the level of the cell membrane exclusively by an as-yet-unknown mechanism? It is accepted that lipid peroxidation is a well-known cause of cellular injury in both animals and plants, and is used as a reliable marker of OS in tissues and cells. Lipid peroxides are derived from polyunsaturated fatty acids; generally they are unstable and decompose to create a broad range of various compounds. These include the highly reactive three-carbon dialdehyde, a compound produced as a byproduct of polyunsaturated fatty acid peroxidation that readily combines with several functional groups on molecules including DNA, proteins, and lipoproteins [55]. Thus, it appears that AKH plays a role in inhibiting lipid peroxidation; however, the mechanism is not properly understood [36].

The role of AKH in the stimulation of an anti-OS response has also been documented by the total antioxidant activity in hemocyte-free plasma of *P. apterus.* The results showed that injection of paraquat alone potentiated an antioxidant response that is significantly elevated upon co-injection of paraquat with AKH. However, AKH injection alone is not capable of inducing antioxidant activity to the levels elicited by paraquat alone or by co-application with paraquat, though it is more enhanced compared to controls [35]. This apparently indicates that there must be additional mechanism(s) by which AKH triggers its antioxidant responses.

It has been suggested recently that AKH also regulates uncoupling proteins (UCPs) in insects [56,57]; however, the mode of action is unclear and the results suggest a complicated mechanism with involvement of other factors. UCPs are usually found in the mitochondrial membrane, where they uncouple the fuel oxidation from ATP synthesis via the electron respiratory chain by directing proton flow into the mitochondrial matrix [58]. One of the uncoupling proteins, UCP4, is probably involved in regulating response to OS: its activation resulted in a decrease in superoxide anion radical production [56]. Nevertheless, a downregulation of UCP4 expression at the mRNA and protein levels was recorded in the beetle *Zophobas atratus* after AKH administration [57]. Furthermore, overexpression of UCP5 in *Drosophila melanogaster* neurosecretory cells that secrete AKH led to starvation-sensitive flies [59]. A recent study that employed CRISPR/Cas9-mediated genome engineering to create AKH and AKH plus adipokinetic hormone precursor-related peptide-(APRP) specific mutants in *D. melanogaster* demonstrated an increased susceptibility to OS induced by direct application of paraquat on the nerve cord in these mutants [60], though the exact mode of action by which AKH signaling facilitates the adaptive response to such a challenge is unclear.

In summary, it is apparent that AKHs comprehensively control the anti-oxidative response, probably by more mechanisms; however, our understanding of the mechanism of action is still far from detailed. More studies are needed to reveal whether the mechanisms are independent or whether they work in cascades involving mutually affecting, positive and negative feedbacks or other principals common in hormonal signaling pathways.

### 5.2. Role of AKH in Oxidative Stress Elicited by Insecticides

Insecticides represent a potent stressor able to elicit various kinds of stress in target insects. Beside their usage in insect pest control, the insecticides are also used in research to evoke experimental stress conditions; this approach is also taken in delving into AKH’s function. There are several reasons for using insecticides for stress research: they are effective in small amounts, often penetrate the cuticle, have an established mechanism of action, and are easy to handle. Using insecticides in the study of modulation of AKH titer was first reported by Singh and Orchard [61], who documented the release of adipokinetic peptides from isolated corpora cardiaca of the locust *L. migratoria* following insecticide application. Later on, Candy [62] recorded a significant release of AKH from the corpora cardiaca and an elevation of its titer in the hemolymph of *Schistocerca gregaria* treated with the insecticide deltamethrin. Also, permethrin treatment of the firebug *P. apterus* resulted in a significant increase of the AKH titer in CNS (including corpora cardiaca) [53] and hemolymph [63]. Further, indirect toxin application—feeding of the Colorado potato beetle, *L. decemlineata* on genetically modified potatoes expressing *B. thuringiensis* toxin or *G. nivalis* lectin—increased the titer of AKH both in CNS and hemolymph [31]. These results clearly indicate involvement of AKHs in the activation of protective mechanisms against insecticidal stress. The increase of AKH level in the corpora cardiaca after treatment was rather variable, while the elevation of AKH in the hemolymph was unequivocally more robust. As mentioned above, these results reflect a low level of correlation between release and biosynthesis of the AKHs [40].

Recently, a group of insecticides—endosulfan, malathion, pirimiphos-methyl, and deltamethrin, which are known to elicit OS in affected insects—was employed to extend our knowledge about the role of AKH in the control of anti-OS reactions [37,38,53]. Accordingly, the insecticides deltamethrin and pirimiphos-methyl significantly reduced both total antioxidant capacity and activity of SOD in *Tribolium castaneum* body [38]. The former result indicates a general reduction of capacity of the defense systems (both enzymatic and non-enzymatic) against OS; the latter suggests a more direct inhibitory effect on the SOD molecules that can be impaired by, for example, protein carbonylation. On the other hand, activity of CAT was significantly enhanced by insecticide treatment (endosulfan, malathion, pirimiphos-methyl, and deltamethrin) of *P. apterus* or *T. castaneum* [37,38], which indicates that overproduction of hydrogen peroxide in the insect body is not exclusively attributable to SOD activity. Further, pirimiphos-methyl and deltamethrin stimulated activity of GST [38], which indicates overproduction of the substrate (primarily peroxides, but not hydrogen peroxide). Data on GST activity in insecticide-treated insects are widely available because this enzyme is under intensive investigation due to its role in the development of insecticide resistance [64]. Elevated levels of GST activity have been reported in organophosphate-, organochlorine-, and pyrethroid-resistant mosquitoes (*Anopheles gambiae*, *A. dirus*, and *Aedes aegypti*) [64], pyrethroid-resistant planthopper (*Nilaparvata lugens*) [65], organophosphate- (including pirimiphos-methyl) resistant maize weevil (*Sitophilus zeamais*) [66], decamethrin-treated mealworm beetle (*Tenebrio molitor*) [67], and many others.

Subsequent results also implied involvement of AKHs in the activation of antioxidant protection mechanisms. All biomarkers of OS impacted by endosulfan, malathion, pirimiphos-methyl, and/or deltamethrin were eliminated/reduced by co-application of the insecticides with AKH, thus the levels of the markers reverted to those observed in respective controls [37,38]. The unequivocal role of AKHs in the anti-OS response was confirmed by experiments in which *T. castaneum* individuals with AKH production deficiency (prepared by RNAi-mediated knockdown) were used [38]. The level of their total anti-oxidative capacity was lower, and the level of GST activity higher than those in beetles producing a normal AKH level. Relevant differences were recorded when pirimiphos-methyl and deltamethrin were applied on these two groups of beetles.

Interestingly, it has been found that the AKH defense reaction against insecticidal stress can also be counter-productive: the co-application of insecticides together with AKH resulted in an increase in insect mortality compared to that induced by the insecticides alone (Table 1). The increase was recorded to be significant for permethrin [53], endosulfan, and malathion [37], or pirimiphos-methyl and deltamethrin [38] applied on *P. apterus* and *T. castaneum*, respectively. The effect was similar for both injection and topical application of agents; however, the maximum effect was recorded for injection of 200 ng of endosulfan and 80 pmol of AKH into the *P. apterus* body: the co-injection of both agents increased the mortality to more than 90% as compared to 30% mortality elicited by injection of endosulfan alone (Table 1). The phenomenon was confirmed by using the AKH-deficient beetles *T. castaneum*, which were more resistant to the insecticide treatment than those with normal AKH production [38]. The mechanism underlying the interaction of AKH with insecticides resulting in enhanced insect mortality is not clear; however, it has been suggested that an increase in insect metabolism following AKH treatment might play a role [53]. In other words, the elevated metabolism resulting from AKH treatment is accompanied by an intensive turnover of metabolites, including insecticides. This can then result in the faster penetration of insecticides into tissues and cells, thereby enhancing their effects. This hypothesis was supported by a determination of the intensity of metabolic activity: the production of carbon dioxide was significantly higher in *P. apterus* firebugs co-treated by endosulfan or malathion with AKH than that in individuals treated by insecticides alone [37]. Similar results were recorded when the bugs were treated with permethrin [53] and/or when the *T. castaneum* beetles were affected by pirimiphos-methyl or deltamethrin; accordingly, the AKH-deficient beetles showed a low level of carbon dioxide production after the insecticide treatment [38]. Interestingly, it has also been found that there were no dramatic differences (just an insignificant increase) in metabolic activity between insects treated with AKH alone compared to untreated controls. This phenomenon, commonly encountered in AKH research, is discussed above (see Section 5.1).

**Table 1:** Effect of insecticides permethrin (P), endosulfan (E), malathion (M), pirimiphos-methyl (PM), and deltamethrin (D), and their co-application with AKH, on mortality of the firebug *P. apterus* and the beetle *T. castaneum*. Increase of mortality—expressed as increase of percentage of mortality after the co-treatment insecticide + AKH as compared to the effect of insecticide alone; mortality ratio—is the ration between the two latter mentioned mortalities; abbreviations: inj—injection, top—topical application, res. film—application by residual film, dip—dipping, AKH-def.—beetles with AKH production deficiency; the numbers in parentheses in the Mortality column represent numbers of repetition (*n*). Statistically significant differences at the 5% level between the insecticide treatment and insecticide + AKH co-treatment were evaluated by one-way ANOVA with the Dunnett’s *post hoc* test (for P, PM and D), and by the Student’s *t*-test (E, M); the differences are labeled by asterisks. Prepared on the basis of the results of our three papers [37,38,54].

Insect Species	Treatment	Mortality (%)	Increase of Mortality (%)	Mortality Ratio
*P. apterus*	P 50 ng inj.	26.6 ± 8.9 (7)	-	–
	P 50 ng inj. + AKH 10 pmol inj.	31.5 ± 13.6 (12)	4.9	1.18
	P 50 ng inj. + AKH 80 pmol inj.	61.0 ± 8.5 * (6)	34.4	2.29
	P 50 ng inj. + AKH 80 pmol top.	41.3 ± 12.1 (8)	14.7	1.55
	P 100 ng inj.	52.0 ± 14.6 (13)	–	–
	P 100 ng inj. + AKH 10 pmol inj.	61.2 ± 2.4 (6)	9.2	1.18
	P 100 ng inj. + AKH 80 pmol inj.	76.1 ± 4.9 * (8)	24.1	1.46
	P 100 ng inj. + AKH 80 pmol top.	82.9 ± 14.4 * (7)	30.9	1.59
	P 400 ng top.	45.4 ± 9.0 (4)	–	–
	P 400 ng top. + AKH 80 pmol top.	73.1 ± 7.2 * (5)	–	1.61
	E 200 ng inj.	30.0 ± 4.0 (4)	–	–
	E 200 ng inj. + AKH 80 pmol inj.	91.5 ± 2.5 * (4)	61.5	3.05
	E 250 ng inj.	57.5 ± 2.9 (4)	–	–
	E 250 ng inj.+AKH 80 pmol inj.	98.7 ± 2.5 * (4)	41.2	1.72
	M 300 ng inj.	25.0 ± 7.1 (4)	–	–
	M 300 ng inj. + AKH 80 pmol inj.	36.2 ± 8.5 (4)	11.2	1.45
	M 450 ng inj.	46.2 ± 10.3 (4)	–	–
	M 450 ng inj. + AKH 80 pmol inj.	73.7 ± 7.5 * (4)	27.5	1.59
	E 450 ng top.	17.5 ± 2.8 (4)	–	–
	E 450 ng top. + AKH 80 pmol top.	31.2 ± 4.7 * (4)	13.7	1.78
	E 1100 ng top.	51.2 ± 4.7 (4)	–	–
	E 1100 ng top. + AKH 80 pmol top.	88.7 ± 4.7 * (4)	37.5	1.73
	M 500 ng top.	15.0 ± 4.0 (4)	–	–
	M 500 ng top. + AKH 80 pmol top.	21.2 ± 4.7 (4)	6.2	1.41
	M 900 ng top.	48.7 ± 4.7 (4)	–	–
	M 900 ng top. + AKH 80 pmol top.	68.7 ± 8.5 * (4)	20.0	1.41
*T. castaneum*	PM 0.53 µg/mL res. film	48.8 ± 2.3 (5)	–	–
	PM 0.53 µg/mL res. film + AKH 35 pmol/μL dip.	71.2 ± 1.5 * (5)	23.3	1.46
	PM 0.53 µg/mL res. film-AKH-def. beetles	26.0 ± 2.5 * (5)	−22.8	0.53
	D 13.92 µg/mL res. film	56.0 ± 2.8 (5)	–	–
	D 13.92 µg/mL res. film + AKH 35 pmol/μL dip.	76.8 ± 2.3 * (5)	20.8	1.37
	D 13.92 µg/mL res. film-AKH-def. beetles	32.0 ± 2.5 * (5)	−24.0	0.57

### 5.3. A Plausible Mechanism of AKH Action in Anti-Oxidative Stress Response

Release of AKHs into the insect blood circulation triggers a cascade of reactions that are initiated by the binding of these neuropeptides to G-protein coupled receptors (GPCRs) located on the membranes of target cells [68,69]. The GPCRs for AKH, which is structurally related to the receptors of the vertebrate gonadotropin-releasing hormone, have been identified and characterized in the fruit fly *D. melanogaster* and the silkworm *Bombyx mori* [68,70,71] as well as in other insect species [72,73,74,75,76]. It has been demonstrated earlier that AKH induced mobilization of nutrients (lipids, carbohydrates, proline) in the target tissues (mostly fat body) is initiated by triggering of a classical intracellular cascade typical for peptide hormone signaling via the adenylate cyclase [77] or phospholipase C [78] pathways, both involving presence of extracellular and/ or intracellular Ca^2+^ [25,79].

**Figure 1:**
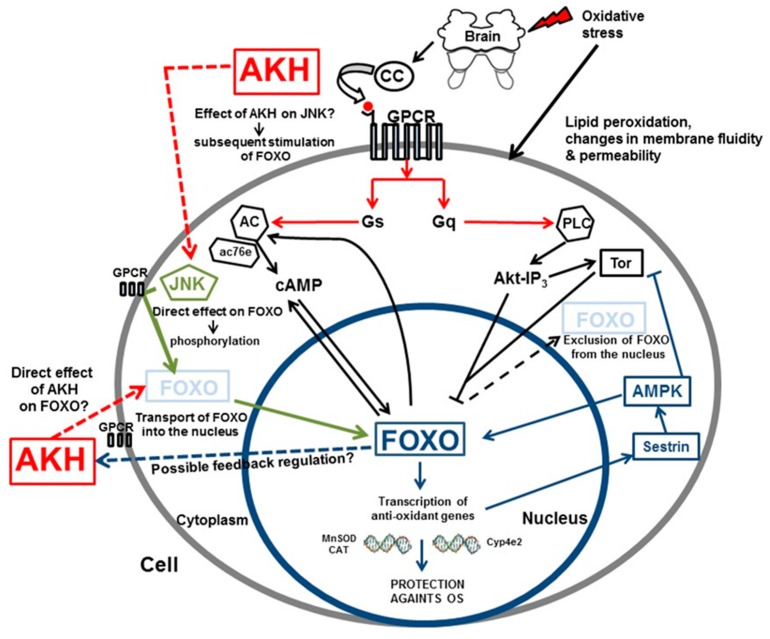
A hypothetical model for AKH action and for the role of FoxO in response to OS in insects. Activation of adenylate cyclase (AC) by AKH through G-protein coupled receptors (GPCRs) leads to an elevation of cAMP, which can directly affect FoxO; FoxO can then feedback and modulate the level of cAMP by direct activation of ac76e. This will induce FoxO localization in the nucleus, where FoxO can act as a transcription factor. Activation of phospholipase C (PLC), on the other hand, leads to elevation in levels of IP3 and protein kinase B (Akt), which leads to degradation of FoxO and its release from the nucleus. Protein kinase B causes phosphorylation of FoxO in the nucleus at the 14-3-3 binding site. This phosphorylation of FoxO masks the nuclear localization signal and prevents nuclear translocation, thereby inhibiting the activities of FoxO. Activation of Jun N-terminus kinase (JNK) under OS conditions directly affects FoxO and leads to its phosphorylation and nuclear translocation, where FoxO can manifest its function in protection against OS. It might also be possible that AKH directly (or indirectly through JNK) stimulates FoxO translocation into the nucleus. FoxO might then have a feedback regulation effect on AKH. FoxO may also activate AMP-activated protein kinase (AMPK) by increasing levels of sestrin in response to stress, which will subsequently lead to inhibition of TOR. TOR is a downstream effector of Akt-IP3. Moreover, AMPK activated by FoxO through sestrin inhibits TOR and at the same time has a positive feedback effect on FoxO. The mechanism by which FoxO confers oxidative-stress resistance most probably runs via the transcriptional uregulation of anti-oxidative enzyme genes e.g., manganese superoxide dismutase (MnSOD), catalase (CAT), and genes encoding cytochrome P450 enzymes (Cyp4e2), or stress responsive proteins such as sestrin (see also corresponding text). Bold arrows represent known pathways whereas dashed arrows represent hypothetical pathways.

On the other hand, only limited information is available on the mechanism of AKH action at the sub-cellular level during OS in insect body. However, a recent investigation on isolated organ cultures of the CNS (brain with corpora cardiaca attached) of *P. apterus* demonstrated an interesting modification of the abovementioned intracellular cascades: the involvement of both protein kinase C and cyclic adenosine 3′,5′-monophosphate (cAMP) pathways which require extra and intra-cellular Ca^2+^ stores [80]. In this study, treatment with various inhibitors of signaling pathways following exposure to stressor (hydrogen peroxide) revealed that the key signaling events are conserved in the manner in which AKH acts. However, further details of the mechanism are still far from clear, but it is expected that identification of specific regulatory mechanisms and downstream effector molecules can possibly provide some insights into the modulation of specific physiological programs that are altered during a situation of OS. Indeed, recently published work using the *Drosophila* model system presented evidence that AKH may primarily employ the Forkhead box class O transcription factor (FoxO) to exert its effect in protection of the organism against OS [81]. The FoxO transcription factors are a family of conserved proteins involved in the regulation of cellular response to various stimuli, such as energy deprivation, stress, and developmental cues. FoxO proteins are also important mediators of the response to OS. In *D. melanogaster dFoxO* proteins are expressed predominantly in fat body [82] and play an important mediatory role in the insulin signaling pathway, which adjusts growth and metabolism to nutrient availability. The insulin signaling pathway and AKH-stimulated cAMP signaling pathway also seem to be linked, or they use similar mechanisms to orchestrate the organism’s response to both nutritional conditions and stress. Findings from the work of Bednářová *et al*. [81] are consistent with the idea that there may be a link between these two signaling pathways or they could act in concert when facing a stress situation. Further, the results are consistent with the conclusions of Süren-Castillo *et al*. [83], who demonstrated that FoxO is required for the activation and expression of the hypertrehalosemic hormone, a member of the AKH-family in cockroaches. As mentioned earlier, in insects AKHs might be the main hormones that counteract the action of insulin. One can hypothesize then there could be a feedback regulation between AKHs and FoxO, which is activated by starvation, and in which the FoxO triggers transcriptional activation elicited by AKHs. On the other hand, under conditions of OS AKHs may trigger FoxO either directly or indirectly to combat OS (Figure 1).

Central to FoxO’s role in regulating transcriptional changes in response to stress is its localization in the cytosol (thereby terminating its transcriptional function) and its subsequent translocation to the nucleus, where it can act as a transcription factor. In *Drosophila*, two major regulatory circuits by which FoxO regulates target of rapamycin complex 1 (TORC1) and subsequently protein kinase B activity have been identified [84]. FoxO can induce transcriptional activation of sestrin, which could lead to elevated levels of the energy sensor protein AMP-activated protein kinase (AMPK), which has an inhibitory effect on the transcript of the *Drosophila* homolog of the target of rapamycin (*dTOR*) [85]. The inhibition of TOR by FoxO is manifested under OS conditions in flies. The sestrins were originally discovered in mammals as antioxidants and are a family of highly conserved proteins [86,87]. Interestingly, they have been shown to activate AMPK, although the precise mechanism is not fully understood [88]. Our hypothesis, mentioned in our recently published study [81], is that AKH could also potentiate the FoxO-Sestrin-AMPK-TOR pathway in its stress responsive role.

FoxO transcription factors have been reported to promote resistance to OS, premature aging, and cellular senescence across species [89]. Further, Jünger *et al*. [90] reported that the adult *dFoxO* mutant flies were hypersensitive to OS conditions: when placed on hydrogen-peroxide-containing food, *dFoxO* mutant flies displayed significantly reduced survival time compared to controls. The authors obtained the same results with paraquat feeding. The *Drosophila* *adenylate cyclase* (*AC*) gene, *ac76e* was identified by Mattila *et al.* [91] as a direct transcriptional target of dFoxO *in vitro* and *in vivo*. They demonstrated that dFoxO-induced activation of AC76E resulted in an increase of cAMP levels, which modulated developmental growth and starvation resistance. Their findings clearly confirm the role of dFoxO as a mediator of cAMP signaling. We suggest that the mechanism by which FoxO activates AC, which results in an increase of the cAMP level, might work by a feedback mechanism as well. This would imply that FoxO transcriptional activity might be increased by a higher level of AC and subsequently of cAMP by imminent action of AKH (Figure 1). It has been demonstrated previously that FoxO and cAMP signaling are involved in the regulation life span and resistance to stress [92,93].

Another kinase that phosphorylates specific sites of FoxO is c-JUN N-terminal kinase (JNK), a member of the mitogen-activated protein kinase family that is activated by stress stimuli [94,95]. Activation of JNK leads to the phosphorylation of FoxO factors and drives the translocation of FoxO into the nucleus, where FoxO is able to exert its function. For instance, recent findings in mouse pancreatic β cells indicate that activation of JNK by OS results in FoxO1 translocation to the nucleus [96]. One of our hypotheses is that during situations of OS AKHs might work in a manner similar to JNK’s and activate FoxO directly by its phosphorylation and subsequent translocation into the nucleus. Moreover, one can also hypothesize that AKH may act indirectly by triggering JNK, or in other words AKH might serve as a mediator of JNK stimulation, which subsequently leads to nuclear translocation of FoxO (Figure 1).

On the other hand, there are other factors influencing FoxO functions. The PI3-protein kinase B pathway is a major upstream signaling module leading to the phosphorylation of FoxO factors and their exclusion from the nucleus [97,98,99]. Across species, FoxO transcription factors have highly conserved phosphorylation sites that are phosphorylated by protein kinase B. This phosphorylation takes place in the nucleus and creates the 14-3-3 binding site to FoxO, which masks the nuclear localization signal and prevents nuclear translocation, thereby inhibiting the activities of FoxO [84]. The energy sensor AMPK is another candidate as a regulator of FoxO function. As mentioned before, FoxO indirectly activates AMPK, which subsequently inhibits TOR activity. The activation of AMPK by FoxO occurs through the transcriptional up-regulation of sestrin [84]. Of note, the energy status of cells also impacts FoxO activity, as AMPK was shown to phosphorylate FoxO and to facilitate its nuclear localization. Thus, AMPK can augment the FoxO-sestrin-AMPK axis by a feed-forward mechanism (Figure 1).

The mechanism by which FoxO confers OS resistance is not yet known. Jünger *et al.* [90] identified several genes encoding cytochrome P450 enzymes (Cyp4e2) as dFoxO target gene candidates. Glauser and Schlegel [99] proposed in their work that genes like *manganase superoxide dismutase* (*MnSOD*) and *CAT* might be employed by FoxO, too, and therefore they partially mediate the protective effect of dFoxO. This suggests that the anti-OS activities of FoxO are mainly attributable to their ability to induce the expression of anti-oxidants genes.

The precise pathway by which FoxO signaling may be activated, and its relationship to AKH, remains hypothetical at this point in time. It is also important to highlight that there might be other players and factors involved, which adds an additional layer of complexity to the whole process of AKH signaling during a situation of OS. Little is known about the genomic targets through which AKH signaling acts. It is plausible that in fruit flies insulin-like peptide signaling and AKH signaling are closely linked [100], and such signaling by AKH might involve FoxO as a mediator, particularly in its role in the anti-OS response. The paracrine and endocrine effects of AKH in response to OS are largely unexplored and might involve multiple targets and genes; the whole pathway might be more complex than is now assumed. Further studies would be necessary to pinpoint the precise mechanism by which FoxO and AKH signaling might interact in maintenance of homeostasis by responding to OS.

### 5.4. Drosophila Melanogaster—An Excellent Model for Anti-Oxidative Stress Response Investigation

The fruit fly, *D. melanogaster*, has so far served as an excellent model system for studying OS resistance pathways [101]. The use of this model organism in research offers numerous advantages and the opportunity for a comprehensive understanding and integration of the different aspects of stress. One of the most important advantages, an evolutionary conservation of the molecular mechanisms of physiological response to stress from *Drosophila* to mammals, makes this model indeed universal. Further, the genome of *Drosophila* has been sequenced [102] and there is a vast array of mutants available at various international centers. The fruit fly serves as a powerful tool to investigate complex pathways since it is genetically tractable and, with the advent of the Gal4/UAS system and recent genome editing tools such as ZNFs, TALENS, and CRISPR/cas9 systems, it is possible to selectively knock out, knock in, or overexpress various genes to understand their functions more precisely. Moreover, *Drosophila* is very easy to breed in laboratory conditions and the experimental treatment of fruit flies is not affected by the ethical and legal concerns associated with higher animals.

In the field of neurohormonal research and particularly with reference to the mode of action of AKH, this model organism would be considered the most appropriate. In *Drosophila* there is a single AKH gene (acronyms: *Dmel/AKH* or *Drome-AKH*) that encodes the peptide hormone precursor of 79 amino acid residues (aa) including the N-terminal signal peptide (22 aa), the active AKH octapeptide, and the 49-aa carboxy-terminal AKH associated peptide (function unknown) [103]. The sequence of the mature Drome-AKH octapeptide is pGlu-Leu-Thr-Phe-Ser-Pro-Asp-Trp-NH_2_, where pGlu is pyroglutamic acid, and Trp-NH_2_ is tryptophan carboxyamide [104]. Similarly, biochemical studies identified a single *Drosophila* AKH receptor (AKHR) [68,70] (see also Section 5.3), which has been functionally analyzed *in vivo* [105,106]. The importance of individual aa in the Drome–AKH octapeptide for the AKHR signaling was recently elucidated; it was shown that the aa at positions 2, 3, 4 and 5 are crucial for receptor activation [69]. The AKHR is located in the plasma membrane of fat body adipocytes [69]. It was shown recently that AKHR uses G protein αq subunit (Gαq) and evokes Ca^2+^ release as a second messenger system in the *Drosophila* adult fat body [107].

Studies in the last decade employed tools for AKH research such as the ablations of corpora cardiaca cells [30,108,109], stimulation of the secretory activity of corpora cardiaca cells by their depolarization [108], mutations of the receptor [105,106,110], overexpression of AKH cDNA [30,105,107,108,111], and AKH RNAi [107,112]. Above all, the null mutant fruit fly in AKH secretion has been prepared by targeted mutagenesis of *Drome–AKH* (*AKH^1^*) using engineered nucleases [113]. The germline targeting of *Drome–AKH* was performed by means of a specific TALEN pair. The *AKH^1^* carries a 3-bp deletion leading to a loss of the second amino acid in the Drome–AKH octapeptide. Similarly, an AKH single mutant (*Akh^A^*) and AKH plus APRP double mutants (*Akh^AP^* and *Akh^SAP^*) were created by CRISPR/Cas9- mediated genome engineering [60]. The AKH-specific mutant (*Akh^A^*) allele represents an AKH-specific in-frame deletion of two C-terminal amino acids of the AKH octapeptide, which leaves the adipokinetic hormone precursor related peptide (APRP) sequence unaffected, but results in an AKH-specific null allele of *Akh* as the AKH^A^ hexapeptide lacks the tryptophan at position 8 that is essential for *Drosophila* AKH receptor activation [69]. The *Akh^AP^* allele carries a 19 bp deletion in the *AKH* region, which causes a frameshift upstream of the APRP coding sequence. A 206 bp deletion in the *Akh^SAP^* allele removes the AKH coding sequence along with the signal peptide sequence and translation initiation side of the prohormone. These new mutants would allow for addressing AKH-specific functions and APRP specific functions in *Drosophila* in future.

*Drosophila* has already been proved to be an excellent model system for investigating several conserved hormonal pathways (e.g., the insulin/insulin like pathway [114,115]); however, only a limited number of studies employ *Drosophila* to elucidate the AKH pathway in regulation of anti-oxidative defense [60,81]. Future research employing this excellent model system should offer deeper insights into the precise signaling pathways activated by AKH in its anti-OS responsive role.

## 6. Other Hormones Controlling the Anti-Oxidative Stress Response in Insects

Besides AKHs, whose role in OS is discussed in the previous sections, other insect hormones have also been considered for their involvement in anti-oxidative protective reactions. They include peptide hormones, ecdysteroids, and juvenile hormones.

### 6.1. Glucagon

Glucagon, is a well-known 29-amino acid peptide hormone playing a critical role in vertebrate glucose metabolism. However, in insects, its role and the role of similar glucagon-like peptides (GLPs) do not seem to be identical to that in vertebrates, despite the fact that there are some indications that GLPs are involved in the regulation of carbohydrate levels in insects, as was documented e.g., in the honey bee *Apis mellifica* [116]. In fact, the role of glucagon in insects is far from clear and has not been explained satisfactorily thus far [21]. Nevertheless, some studies indicate that glucagon/GLPs are included in defense against insect OS [117,118]. The effect of mammalian glucagon has been evaluated in the firebug *P. apterus* [117], wherein its injection into the body elicited an antioxidant response by causing a significant decline in protein nitrotyrosine and protein carbonyl levels, and elevation of GSH. Further, co-injection of glucagon together with paraquat partly eliminated OS markers evoked by injection of paraquat alone, and reverted them to the levels in untreated controls. Interestingly, glucagon action at least in this anti-oxidative stress response seems to be AKH independent, because no significant changes in AKH level in the *P. apterus* body were recorded after the glucagon injection.

The function of glucagon and GLPs in stress responses has been summarized recently [118]. The authors suggested the hypothetical mechanism of not only glucagon, but also glucagon-like peptides (GLP-1, GLP-2) and AKH release and action in stress situation in both vertebrates and insects. The signal of those hormones is mediated via GPCR and cAMP and Ca^2+^ secondary messenger system similarly to AKH signaling (for details see Section 5.3).

### 6.2. Corazonin

Corazonin is an 11-amino acid neuropeptide highly conserved throughout insects that was originally characterized as a cardioaccelerating peptide stimulating the heartbeat in the cockroach *Periplaneta americana* [119]. However, later on corazonin has been shown to be multifunctional hormone affecting a wide range of biochemical, physiological, and behavioral functions in insects (color changing, pigmentation, phase dimorphism, ecdsysis behavior, circadian rhythms, heat and cold tolerance, metabolism, spinning) [120]. Corazonin function in stress responses have been hypothesized by Veenstra [121]. Accordingly, it has been proven that ablation of corazonin neurons confers resistance to metabolic, osmotic, and OS in *D. melanogaster*; nevertheless, no change of corazonin transcript expression under OS was reported [122]. The survival under OS conditions was significantly prolonged in corazonin-deficient flies as compared to the controls, and the survival extension was higher in females than in males.

### 6.3. Prothoracicotropic Hormones

These neuropeptides appear in two main forms, a small prothoracicotropic hormone (PTTH) or bombyxin (5–19 kDa, [123]), and a large PTTH (24–30 kDa, [124]) that mainly controls ecdysteroid synthesis in the prothoracic gland [125]. Recently, it has been suggested that PTTH is also involved in processes of stress adaptation by regulating development and morpho-physiological changes. The secretion and size of brain neurosecretory cells producing PTTH significantly increased in *Lymantria dispar* larvae fed on an unsuitable host plant, eliciting OS conditions [126]. The stressed larvae also exhibited increased activity of SOD and a higher amount of GSH in the midgut. On the other hand, CAT activity remained unchanged by diet, which the authors explained by possible substitution of the CAT function by ascorbate peroxidase.

### 6.4. Ecdysteroids

These steroid hormones play a pivotal role in insect development and reproduction and control a number of accompanying processes. Moreover, it has recently been revealed that 20-hydroxyecdysone (20-HE), one of the most important ecdysteroids, showed a potent anti-oxidative activity: the OS activity of paraquat was suppressed by co-injection with 20-HE in *P. apterus* [127]. 20-HE curbed the formation of protein carbonyls and lipid peroxidation, enhanced the reduced GSH level, improved fluidity in microsomal membrane, and increased the γ-glutamyl transpeptidase activity in the brain. At the organismic level, 20-HE eliminated three harmful effects elicited by injection of paraquat: the suppression of female fertility, the disappearance of certain hemolymph proteins, and the reduction of adult life span in both *P. apterus* sexes. In the fruit fly, *D. melanogaster*, 20-HE has been shown to induce methionine sulfoxide reductase A [128], which is associated with resistance to hydrogen peroxide. Methionine residues in proteins are sensitive to oxidation when subjected to reactive oxygen and nitrogen species (ROS/RNS) [129]. The reduction of the oxidized methionine form is catalyzed by methionine sulfoxide reductase [130] and expression of this enzyme is controlled by ecdysone through the ecdysone receptor (EcR-UPS) [131]. Overexpression of methionine sulfoxide reductase is associated with elevated protection against OS, while its knockdown results in higher susceptibility to OS [132,133].

Moreover, insect 20-HE has been found to protect mammals against cerebral ischemia injury by inhibiting production of ROS/RNS and modulating OS-induced signal transduction pathways [134]. Treatment of B35 rat neuroblastoma cells with hydrogen peroxide led to OS-induced apoptosis, mitochondrial membrane potential dissipation, neuronal injury, generation of intracellular ROS/RNS, decrease of cellular anti-oxidant potential, elevation of Ca^2+^ intracellular concentration, and increase of lipid peroxidation, all of which were significantly eliminated by 20-HE. The neuroprotective effect of 20-HE was also confirmed *in vivo*. Further, in rats, 20-HE significantly decreased infarct volume and the neurological deficit score, restored anti-oxidant potential, and eliminated the MDA (malondialdehyde) formation increase and TUNEL- and cleaved caspase-3-positive cells in the cerebral cortex of middle cerebral artery occlusion [134].

Similarly, the steroid vertebrate hormones, estrogens, and related compounds with a phenolic A-ring were shown to control not only reproduction but also anti-oxidative response. They inhibited the cholesterol oxidation and peroxidation of polysaturated fatty acids (MDA formation and diene conjugation) in the microsomes, lipoproteins, and other components of the biological system [135].

### 6.5. Juvenile Hormones

Juvenile hormones (JHs), insect multifunctional terpenoids, are predominantly involved in the control of developmental events and reproductive processes. In juvenile stadia JHs prevent premature commencement of insect metamorphosis, and in adult females they support reproduction by hormonally controlled gene expression of vitellogenins. JHs are synthesized and secreted in/from the corpora allata, a small paired (rarely fused) organ close to the brain. Recent studies have implicated that lack of JH may confer resistance to OS. The knockout of corpora allata in *Drosophila*, leading to lack of JH, resulted in higher resistance to OS [136]. The elimination of JH synthesis extended the survival of flies exposed to hydrogen peroxide, but did not increase the survival of starving flies. As anticipated, the survival times returned to those in controls (wild-type flies) when the knockout flies were treated with JH analogue methoprene. Moreover, another JH analogue, pyriproxyfen, induced OS in the waxmoth *Galleria mellonella*, because the activity of anti-oxidative enzymes CAT and SOD increased after the pyriproxyfen application [137]. Similarly, pyriproxyfen increased the activity of enzymes CAT and GST, as well as the accumulation of MDA and GSH, in larvae of *S. littoralis* [138].

In *Bombyx mori*, an interesting hormonal cooperation in regulation of GST gene expression has been recorded. The expression was downregulated by JH-analogue and upregulated by 20-HE [139]. This phenomenon represents a typical example of coordinated function of antagonistic hormones.

JHs are also involved in the control of anti-oxidative reactions albeit indirectly via the regulation of synthesis of biologically important proteins like vitellogenins [140] and transferrin [141,142].

Primarily, vitellogenins are synthesized in specialized cells of the fat body or in certain insect species directly in ovarioles by a complicated process controlled by several hormones [143]; among them, the JHs in most insect species play a crucial role. Vitellogenins represent the main source of energetic and building components for the developing embryo in the insect egg. Besides their function in reproduction, vitellogenins have been proven to be a preferred target of protein carbonylation compared with other proteins of hemolymph [144]. Thus, it has been proven that vitellogenins protect the worker bees (*Apis mellifera*) against the oxidative damage [140]. When treated with paraquat the survival of high-vitellogenin bee phenotypes was significantly higher than that in low-vitellogenin ones. These data show that there is a feedback between vitellogenin activity and OS resistance in bees; this could help explain why vitellogenins are abundant in long-lived workers and queens.

The main function of transferrin is transport of iron, required for a broad number of metabolic processes including electron transfer and oxygen transport; iron also plays a crucial role in the Fenton reaction (see above), in which the very reactive hydroxyl radical is generated [145]. It is supposed that insect transferrin is regulated by JH activities including vitellogenins. It has been shown that JH treatment suppressed transferrin gene expression in the fat body of the cockroach *Blaberus discoidalis* [141]. Likewise, in the mosquito *Aedes aegypti*, methoprene JH analogue, has been revealed to suppress the stimulation of transferrin messages after blood feeding [142]. The reason for such suppression is far from clear: the transferrin gene could be a target of transcription factors of edcysteroid-induced homologues and a negative effect of JH on them could play some role [142].

## 7. Conclusions

Our knowledge about oxygen metabolites and the complex signaling pathways leading to an activation of antioxidant defenses has increased enormously within the last several decades. There is a plethora of literature available about ROS and their mode of action, and about defense reactions against them. Several neurodegenerative diseases including Parkinson’s and Alzheimer’s disease are associated with the development of OS and have been intensively studied. Accordingly, most of the data were obtained on vertebrates; however, a set of papers from the last several years demonstrated that insects represent an important model for the OS research. The insect model is interesting not only theoretically but also for a number of practical reasons that include e.g., possible development of new methods of insect pest control. Further, the results from insects are very often universal and can contribute to elucidation of crucial theoretical problems of OS in animals generally including human beings. The apparent involvement of adipokinetic hormones in the control of anti-OS defense reactions is a typical example of endocrine regulation of such a response. The mechanism(s) of AKH action in such events, of which understanding is necessary for detailed elucidation of the hormonal role, has been revealed only partially and is still poorly understood. However, intensive research, predominantly on the *Drosophila* model, can bring interesting data in the near future. Currently, the *Drosophila* mutants created with the breakthrough genome engineering techniques with AKH- or AKH-receptor deficiency, with AKH over-production and with curbed, non-functional, or deficient factors of anti-OS reaction are easily available. These constitute a very powerful tool for a significant shift in our theoretical and practical knowledge not only in insects, but also for higher animals.

## Abbreviations

AC: adenylate cyclase; *ac76e*: *Drosophila* adenylate cyclase gene; AKH: adipokinetic hormone; AKHR: adipokinetic hormone receptor; AMPK: AMP-activated protein kinase; APRP: adipokinetic hormone precursor related peptide; CAT: catalase; dTOR: target of rapamycin; FoxO: Forkhead box class O; GSH: glutathione; GST: glutathione-S-transferase; JH: juvenile hormone; JNK: c-JUN N-terminal kinase; MDA: malondialdehyde; OS: oxidative stress; PTTH: prothoracicotropic hormone; RNS: reactive nitrogen species; ROS: reactive oxygen species; SOD: superoxide dismutase.
